# New Inflammation-Related Biomarkers during Malaria Infection

**DOI:** 10.1371/journal.pone.0026495

**Published:** 2011-10-20

**Authors:** Fikregabrail Aberra Kassa, Marina Tiemi Shio, Marie-Josée Bellemare, Babacar Faye, Momar Ndao, Martin Olivier

**Affiliations:** 1 Department of Microbiology and Immunology, McGill University, Montréal, Canada; 2 Centre for the Study of Host Resistance, the Research Institute of McGill University Health Centre, Montréal, Canada; 3 Department of Parasitology and Mycology, Faculty of Medicine, Cheikh Anta Diop University, Dakar, Sénégal; 4 National Reference Centre for Parasitology, Montreal General Hospital, Montréal, Canada; Institut national de la santé et de la recherche médicale - Institut Cochin, France

## Abstract

Malaria is one of the most prevalent infectious diseases worldwide with more than 250 million cases and one million deaths each year. One of the well-characterized malarial-related molecules is hemozoin (HZ), which is a dark-brown crystal formed by the parasite and released into the host during the burst of infected red blood cells. HZ has a stimulatory effect on the host immune system such as its ability to induce pro-inflammatory mediators responsible for some of the malaria related clinical symptoms such as fever. However, the host serum proteins interacting with malarial HZ as well as how this interaction modifies its recognition by phagocytes remained elusive. In the actual study, using proteomic liquid chromatographic mass spectrometry (LC-MS/MS) and immunochemical approaches, we compared the serum protein profiles of malaria patients and healthy individuals. Particularly, we utilized the malarial HZ itself to capture serum proteins capable to bind to HZ, enabling us to identify several proteins such as apolipoprotein E (ApoE), serum amyloid A (SAA), gelsolin, complement factor H and fibrinogen that were found to differ among healthy and malaria individual. Of particular interest is LPS binding protein (LBP), which is reported herein for the first time in the context of malaria. LBP is usually produced during innate inflammatory response to gram-negative bacterial infections. The exact role of these biomarkers and acute phase responses in malaria in general and HZ in particular remains to be investigated. The identification of these inflammation-related biomarkers in malaria paves the way to potentially utilize them as diagnostic and therapeutic targets.

## Introduction

Malaria is one of the major public health problems worldwide affecting more than 250 million people causing over one million deaths each year. Caused by the *Plasmodium* parasite and transmitted through the bite of an infected female *Anopheles* mosquito, malaria still remains to be a major public health burden particularly to the developing nations [1].

In uncomplicated malaria cases, some of the malaria symptoms include fever, chills, sweats, headache, nausea, vomiting and general malaise. Severe *P. falciparum* mediated malaria, however, is characterized by cerebral malaria, severe anemia, renal failure, metabolic acidosis, and hyperparasitemia, where more than 5% of the red blood cells (RBC) are infected by the parasites [2]. Severe malaria, which is a complex multi-system disorder, has clinical similarities with sepsis. For example, metabolic acidosis, which is the excessive acidity in the blood and tissue fluids, is also observed in severe malaria patients [3].

During malaria infection, several inflammatory mediators are involved. The presence of high levels of pro-inflammatory mediators such as TNF-α, IL-1β and IFN-γ is correlated with severe malaria [4]. Furthermore, there is a marked increase in plasma concentrations of adhesion receptors such as ICAM-1 and E-selectin [5]. In addition to cytokines, others biomarkers have been used to discriminate cerebral malaria from uncomplicated malaria, such as serum angiopoietin-1 and -2 (ANG-1 and ANG-2). The levels of ANG-1 significantly decrease while ANG-2 increases in cerebral malaria patients [6], [7]. Moreover, ANG-2, which is the angiogenic factor modulating endothelial activation, is significantly elevated in severe malaria patients [8].

Elevated levels of serum and cerebrospinal fluid apoptotic factors such as IP-10 (INF-inducible protein 10 KDa), IL-1ra, sTNF-R1, sTNF-R2 and sFas were also correlated with cerebral malaria-associated mortality in children [9]. Peripheral levels of IL-10, TNF-α and ferritin were elevated in inflammatory placental malaria whereas the level of leptin showed a marked decrease [10]. In this line of thought, discovery of biomarkers could help to discriminate malaria severity, as well as their role in the development of malaria-related pathologies in conjunction with parasite bi-product such as hemozoin (HZ).

During its intraerythrocytic stage, the obligate intracellular malaria parasite utilizes hemoglobin of the RBCs. This hemoglobin proteolysis results in the production of toxic free heme and the parasite developed a heme detoxification mechanism that results in the formation of heme dimmers, called HZ [11]. Each erythrocytic cycle in a malaria patient with 1–10% parasitemia is believed to produce 0.2–2 g of hemozoin [12]. During malaria infection, HZ is released into the circulation with the merozoites during the rupture of infected RBCs and it induces the production of several proinflammatory molecules *in vitro* and *in vivo* [13].

When considering the physicochemical characteristics of HZ crystal, it has been shown to bind lipids, DNA and proteins. HZ has also been produced synthetically, showing the same biological and chemical characteristics as the native one [14]. When it is released into the bloodstream, it comes in contact with several host serum proteins, however, whereas preliminary analysis revealed that native HZ prior to its released in circulation is solely covered by globin fragments (Bellemare et al., unpublished data), the identity of these proteins is still unrevealed.

In the present work, we used the synthetic HZ to capture and identify HZ-binding serum proteins, which can also serve as to discover unique biomarkers related to malaria context. Our analysis have permitted to identify 42 proteins binding to HZ, among which we confirm the presence of previously reported biomarkers such as serum amyloid A (SAA) and gelsolin. In addition, we identified new malaria biomarkers such as LPS binding protein (LBP), apolipoprotein E (ApoE) and alpha-1-antitrypin as well as proteins that are absent in the malaria sample such as clusterin and complement B. Identification of new malaria biomarkers during malaria infection permit us to identify a specific protein profile signature that could be useful for the development of rapid diagnostic tests, and further our understanding in regard to the development of inflammatory-related malaria pathologies.

## Materials and Methods

### Serum samples

Individuals with positive blood smear for P. falciparum were eligible for enrollment. The institutional ethics committee at the Parasitology and Mycology Service of Faculty of Medicine, Cheikh Anta Diop University, Dakar, Senegal granted ethics approval. Written and verbal consent were obtained prior to the experiments. Written informed consents were obtained from the patients or their legal guardians and in the cases where written consents were not possible verbal consents were obtained. Venous blood samples were collected and serum derived from patient blood was immediately frozen, shipped on dry ice, and maintained at −80°C until use at the National Reference Center for Parasitology of the Research Institute of McGill University Health Center, Montréal, Canada. The institutional review board of McGill University approved the study. The sera used were thawed on ice and re-frozen a maximum of two times.

### Hemozoin preparation

HZ was prepared according to [15]. Briefly, hemin (0.8 mmol, 500 mg) was dissolved in degassed NaOH (0.1 M, 100 mL) for 30 min, with mild stirring. Propionic acid (4 ml) was added drop-wise over a 20 min period until a pH of about 4 was achieved. The mixture was allowed to anneal at 70°C for 18 hours. Then the following washes were performed: Three NaHCO_3_ (0.1 M) washes for 3 hours alternating each ones with dH_2_O. Finally, methanol and dH_2_O were used for washing 3 times alternatively. The sample was then thoroughly dried in a vacuum oven overnight over phosphorous pentoxyde. All synthetic hemozoin samples were fully characterized by X-ray powder diffraction (XRD), field emission gun scanning electron microscopy (FEG-SEM), and infra-red spectroscopy (ATR-FTIR).

### Coating hemozoin with serum

To identify serum proteins interacting with HZ, we used at least 8 individual serum samples from mild malaria patients, severe malaria patients, or healthy individuals. Hundred micrograms of HZ was coated with 10% serum in the presence of MEM medium (Gibco), with a final volume of 100 µl. Serum proteins were allowed to bind to HZ by incubating them at 37°C for 30 min. Unbound proteins were removed by washing with 500 µl of phosphate buffered saline (PBS) at 4500×*g* for 5 min. The washing and centrifugation was repeated four times to ensure the maximal removal of unbound proteins. After the last wash, each sample was subject to SDS-PAGE followed by silver staining or Western blotting analysis; and mass spectrometry analysis.

### Silver staining

HZ-bound proteins were resolved by standard 12% SDS-polyacrylamide gel and subsequently visualized by silver staining as described by [16] with slight modification. After electrophoresis, the gel slab was first fixed in 40% methanol/5% acetic acid for 20 min followed by washing with 30% methanol for 10 min. The washing was repeated with water for 10 min to remove the remaining acid. Gel sensitization was achieved by 1 min incubation in 0.02% sodium thiosulphate, followed by 2 washes with distilled water for 1 min each. The gel was then submerged in 0.2% silver nitrate solution and incubated for 30 min at RT. After incubation, the silver nitrate was discarded; the gel slab was rinsed twice with water for 1 min and developed in 37% formaldehyde; 3% sodium carbonate and 0.001% Sodium thiosulphate solution. When the desired intensity of staining was achieved, development was stopped by discarding the reagent and adding 3% TRIS base/2% acetic acid solution for 30 min followed by 1% acetic acid storage solution. All the solutions used were prepared in water and the gel slab was shaking in orbital shaker in all the described steps.

### Western blotting and LPS binding protein (LBP) ELISA

SDS-polyacrylamide gel resolved HZ-bound proteins were transferred to PVDF membranes. After blocking, membranes were immunoblotted with anti-SAA (Santa Cruz Biotechnology, CA), Apo E (Millipore), albumin (Santa Cruz Biotechnology, CA), and LBP (Santa Cruz, CA) antibodies and appropriate secondary antibody conjugated with HRP (from Sigma). Total serum LBP levels (without HZ binding) were determined using commercially available human LBP ELISA kit (HyCult Biotechnology) according to the manufacturer's instruction.

### Liquid chromatography-mass spectrometry (LC/MS/MS)

#### In-solution protein digestion

Protein-coated HZ crystals were subjected to a manual in-solution digestion before analysis by mass spectroscopy at the McGill University and Genome Quebec Innovation Center. Briefly, each tube containing HZ crystal-adsorbed protein solutions were air-dried (SpeedVac). Pellets were resuspended in a 50 mM ammonium bicarbonate buffer (pH 7.8) supplemented with 6 M urea for the proteins to be denatured. One hundred mM DTT in 50 mM ammonium bicarbonate buffer was then added to each tube to a final concentration of 10 mM DTT. Samples were incubated for 30 min for 37°C then cooled for 5 min at RT. After a quick spin, 300 mM iodoacetamide in 50 mM ammonium bicarbonate buffer was added to a final concentration of 27.5 mM iodoacetamide and incubated for 30 min at RT in the dark. The reduced and alkylated samples were diluted to a final concentration of 1.5 M urea with 50 mM ammonium bicarbonate buffer. Digestion was performed by the addition of trypsin at a ratio of 1∶25 (w/w) protease∶protein. After an overnight incubation at 37°C, the reaction was quenched by the addition of formic acid to a final concentration of 1%. Samples were then cleaned using Zip Tip C18 before mass spectrometry analysis.

#### Mass spectrometry

Extracted peptides were subjected to mass spectrometry analysis as subsequently described. Eluted peptides were injected onto a 300 µm×5 mm Zorbax C18 trapping column and peptides were subsequently resolved on a 10 cm×75 micron PicoFrit column containing BioBasic C18 packing. Peptides were eluted from the column with a 30 minutes gradient of 10–95% acetonitrile (v/v) containing 0.1% formic acid (v/v) at a flow rate of 200 nl/min using an Agilent 1100 series NanoHPLC system (Mississauga, ON). Eluted peptides were electrosprayed as they exited the column. Mass spectrometric data were acquired on a QTRAP 4000 (SCIEX/ABI, Concord, ON) using the Information Dependent Analysis (IDA) feature of Analyst 1.4.1 software (ABI, Foster city, CA). Precursor ion selection for subsequent tandem ms fragmentation analysis was done using predefined IDA criteria. Briefly, up to three doubly, triply or quadriply charged ions of intensity greater than 2×10^6^ counts per second (cps) from each enhanced-ms survey scan were selected for passage into a collision cell. Collision-induced dissociation was facilitated by collision with nitrogen gas; fragment ions were trapped in Q3 and scanned. Three enhanced-product ion scans (EPI) at a speed of 400 amu/second from 70 to 1700 m/z were averaged for each selected precursor ion. Accumulation time used for EPI scans was 20 ms with Q0 trapping activated. MS/MS raw data were transferred from the QTrap 4000 linear ion trap mass spectrometer to a 50 terabytes server and automatically manipulated for generation of peak lists by employing Distiller version 2.1.0.0 (http://www.matrixscience.com/distiller.html) software with peak picking parameters set at 1 as for Signal Noise Ratio (SNR) and at 0.3 for Correlation Threshold (CT).

#### Protein database searching

The peak-listed data was then searched against a copy of the Universal Protein Resource (UniPort) database (March 03, 2008) by using Mascot (Matrix Science, London, UK; version 2.1.4.04) and X! Tandem (http://www.thegpm.org version 2007.01.01.1). Mascot was set up to search the *Homo sapiens* (taxon ID 9606) database (71371 entries; 28450293 residues) assuming the digestion enzyme trypsin. X! Tandem was set up to search a subset of the *Homo sapiens* (taxon ID 9606) also assuming trypsin. Mascot and X! Tandem were searched with a fragment ion mass tolerance of 0.80 Da and a parent ion tolerance of 1.5 Da. Iodoacetamide derivative of cysteine was specified in Mascot and X! Tandem as a fixed modification. Oxidation of methionine was specified in Mascot as a variable modification. Pyro-glu from Q of glutamine, deamidation of asparagine and oxidation of methionine were specified in X! Tandem as variable modifications.

#### Protein identification

Scaffold (version 2, Proteome Software Inc., Portland, OR) was used to validate MS/MS based peptide and protein identifications. Peptide identifications were accepted if they could be established at greater than 95% probability as specified by the Peptide Prophet algorithm [17]. Protein identifications were accepted if they could be established at greater than 90% probability and contained at least 1 identified peptide. Protein probabilities were assigned by the Protein Prophet algorithm [18]. Proteins that contained similar peptides and could not be differentiated based on MS/MS analysis alone were grouped to satisfy the principles of parsimony.

### Blast2GO analysis

Bioinformatics analysis was carried out using Blast2GO, a Gene Ontology (GO) annotation, visualization and analysis software (version 1.2.7 http://web3.vs160142.vserver.de). The analysis consists of three sequential steps: basic local alignment search tool (BLAST), mapping and annotation.

#### Blast

The sequences proteins identified by the mass spectrometry were retrieved from Universal Protein Resource (UniProt) database (www.uniprot.org) and were saved as fasta files, since the blast server accepts only fasta-formatted protein sequences as input queries. The protein input queries were blasted against the NCBI Basic Local Alignment Search Tool (BLAST) database http://www.ncbi.nlm.nih.gov/BLAST/to find similar sequences. The input parameters used were as follows: BLAST database, NCBI nr; number of BLAST hits requested for each query, 20; BLAST expectValue (i.e. eValue), 1e-3; BLAST program, blastp; Blast Mode: QBlast-NCBI; HSP length cutoff: 33.

#### Mapping

Mapping was performed using Blast2GO to obtain Gene Ontologies (GO) for hits retrieved by the Blast step. The mapping step searches various databases and over 2 million gene products to identify and fetch GO terms.

#### Annotation

The annotation procedure selects the GO terms from the GO pool obtained by the mapping step and assigns them to the query sequences, using the Annotation Rule. The annotation parameters were: Pre-eValue-Hit-Filter, 6; Pre-Similarity-Hit-Filter, 30; Annotation Cut-Off, 55; GO-Weight, 5.

### Statistical analysis

Data was analysed using GraphPad Prism 5.0. Unpaired t-test was used for the comparison of two parameters. For the comparison of more than two parameters, one-way ANOVA was employed followed by Bonferroni's multiple comparison test. Means were considered significant for P-values <0.05.

## Results

### Sample categorization and visualization of hemozoin-binding proteins

**Figure 1:**
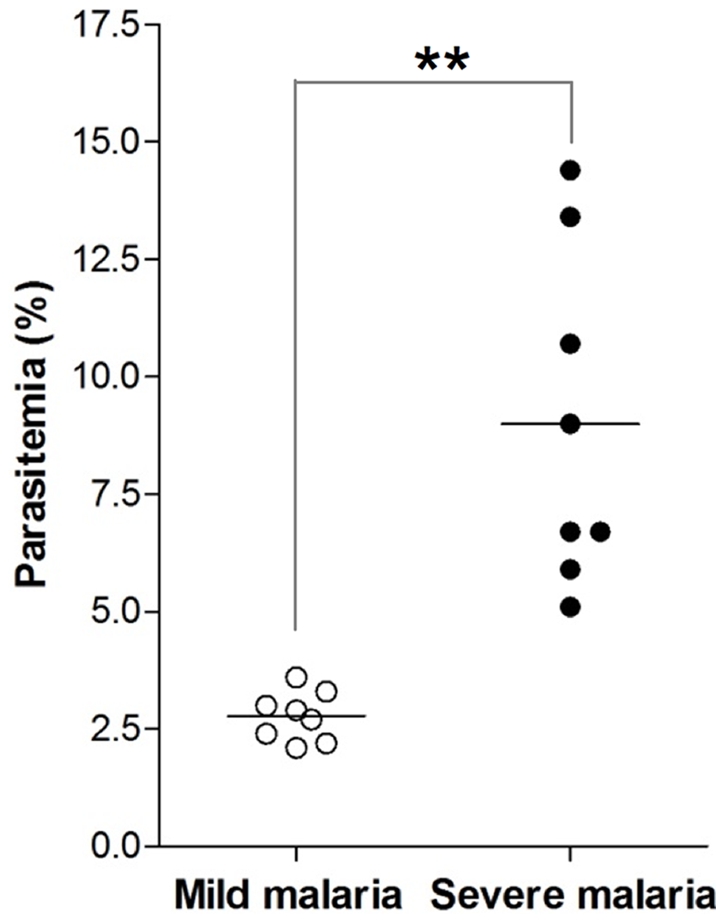
The level of parasitemia (%) of the malaria patients. Malaria patients were grouped into mild and severe based on the level of parasitemia (%). Patients with levels below 5% were grouped into mild and above as severe. Each data point represents individual patient. Sera from eight mild malaria and eight from severe malaria patients were used to identify hemozoin-binding proteins. **P = 0.0002.

Malaria complications can be divided into severe and mild according to the parasitemia level. In the present work, patient serum samples used were classified into two groups: mild and severe malaria. The classification is based on the level of parasitemia, taking 5% as a cutoff value, as shown in Figure 1. Sera from non-infected individuals were used as control (healthy group).

**Figure 2:**
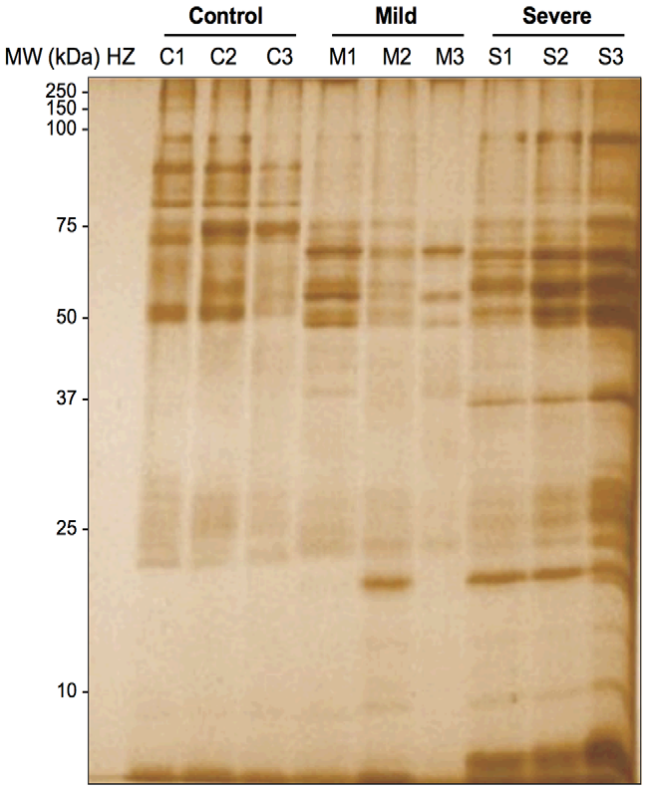
Silver-stained gel showing the profile of hemozoin-binding proteins. Hemozoin was coated with serum from malaria patients (mild and severe) and healthy individuals. The protein-coated hemozoin crystal was subjected to 12% SDS-PAGE and silver-stained. The protein profiles were different among the groups. The hemozoin crystal alone (HZ), used as a control, is shown in the first lane. C1–C3: sera from healthy individuals; M1–M3 sera from mild malaria; S1–S3 sera from severe malaria patients.

In order to visualize the profile of proteins capable to bind to HZ, a synthetic HZ was incubated with serum samples from healthy individuals and malaria patients, and the protein-coated HZ crystal was run onto gel followed by silver staining. As shown in Figure 2, the protein profile of healthy individuals differs substantially from the malaria patients suggesting that proteins adhering to HZ are in part clearly different.

### Identification of malaria biomarkers associated with hemozoin

In order to identify the proteins, the protein-coated HZ was eluted, separated by liquid chromatography (LC) and detected using LC-coupled tandem mass spectrometry (LC-MS/MS). Database searching (Mascot and X! Tandem) was performed for protein identification, as outlined in the Materials and Methods section.

We used the Scaffold 2 Proteome software to visualize, validate, and analyze the identified HZ-binding proteins. A total of 75 proteins were identified. A 95% peptide identification probability and 90% protein identification probability were used as cutoffs for Scaffold. This analysis yielded 42 proteins, excluding the others identified with lesser probability. The complete list of the identified HZ-binding proteins along with their accession number, molecular weights and the mean ± SEM of the normalized spectrum count are shown in Table 1. The identified proteins include lipid binding/transport proteins, acute phase proteins, immunoglobulins, and complement component proteins. HZ-binding proteins were not significantly different between the mild and severe malaria. However, 24 from the 42 identified proteins were significantly different from control and malaria groups. The comparison was done based on the normalized spectrum counts (i.e. spectrum counts divided by the total number of filter-passing peptide identifications from each phase), calculated by Scaffold software and further statistic analysis. Some specific examples of malaria biomarkers bound to HZ included LPS binding protein (P18426), apolipoprotein E (P02649), hemoglobin subunit alpha (P69905), serum amyloid A (P02735) and complement factor H (A5PL14).

**Table 1:** Hemozoin-binding Proteins identified by LC-MS/MS.

Protein name		Proteinaccessionnumber	Swiss-Prot accession number	MW(kDa)	Normalized spectrum count			P-value
					Control(n = 7)	Mildmalaria (n = 8)	Severemalaria (n = 8)	
					Mean ± SEM	Mean ± SEM	Mean ± SEM	
1	Alpha-1-antitrypsin	A1AT_HUMAN	P01009	47	2.33±0.90	8.66±2.40	12.21±2.21	0.0049*
2	Alpha-2-macroglobulin	A2MG_HUMAN	P01023	163	0.57±0.20	0.70±0.53	1.82±0.74	0.3512
3	Apolipoprotein A-I	APOA1_HUMAN	P02647	31	21.30±5.62	13.49±2.03	14.65±1.27	0.0864
4	Apolipoprotein A-II	APOA2_HUMAN	P02652	11	3.96±1.40	1.60±0.91	4.67±1.27	0.6077
5	Apolipoprotein A-IV	APOA4_HUMAN	P06727	45	1.70±0.35	0.65±0.37	0.64±0.34	0.0251*
6	Apolipoprotein B	C0JYY2_HUMAN	C0JYY2	516	4.97±2.32	9.20±2.52	6.63±2.10	0.3210
7	Apolipoprotein C-II	APOC2_HUMAN	P02655	11	0.92±0.39	0	0.25±0.16	0.0096*
8	Apolipoprotein C-III	APOC3_HUMAN	P02656	11	0.92±0.16	0.79±0.24	0.30±0.20	0.1850
9	Apolipoprotein E	APOE_HUMAN	P02649	36	3.68±0.72	8.51±1.13	5.41±1.06	0.0270*
10	Apolipoprotein L1	APOL1_HUMAN	O14791	44	0	0.47±0.23	0.32±0.22	0.1109
11	Clusterin	CLUS_HUMAN	P10909	52	3.15±0.30	0	0	<0.0001*
12	Complement C1q subunit C	C1QC_HUMAN	P02747	26	1.19±0.57	0.34±0.22	0	0.0217*
13	Complement C3	CO3_HUMAN	P01024	187	5.50±1.21	1.85±0.38	2.26±0.59	0.0014*
14	Complement C4-B	CO4B_HUMAN	P0C0L5	193	3.63±1.11	0	0.20±0.20	<0.0001*
15	Complement component C8	CO8B_HUMAN	P07358	67	0.18±0.18	0.50±0.35	0.28±0.18	0.5153
16	Complement component C9	CO9_HUMAN	P02748	63	0.50±0.35	0	0	0.0351*
17	Fibrinogen alpha chain	FIBA_HUMAN	P02671	95	7.11±2.71	14.21±2.53	15.45±2.25	0.0198*
18	Fibrinogen beta chain	FIBB_HUMAN	P02675	56	1.28±0.53	5.40±1.46	4.56±1.18	0.0177*
19	Fibrinogen gamma chain	FIBG_HUMAN	P02679	52	4.57±1.89	9.55±0.99	7.93±0.93	0.0170*
20	Fibronectin	FINC_HUMAN	P02751	263	0.68±0.50	0	0	0.0472*
21	Gelsolin	GELS_HUMAN	P06396	86	3.90±1.97	0.83±0.45	0.58±0.32	0.0256*
22	Hemoglobin subunit alpha	HBA_HUMAN	P69905	15	0.14±0.14	0.35±0.35	2.04±0.96	0.2192
23	Hemoglobin subunit beta	HBB_HUMAN	P68871	16	0.25±0.25	0.72±0.55	2.72±0.90	0.1116
24	Histidine-rich glycoprotein	HRG_HUMAN	P04196	60	4.15±0.75	1.64±0.49	3.87±1.07	0.2154
25	Ig gamma-1 chain C region	IGHG1_HUMAN	P01857	36	2.05±0.61	0.66±0.25	1.27±0.33	0.0454*
26	Ig gamma-3 chain C region	IGHG3_HUMAN	P01860	41	0.82±0.33	0.13±0.13	0	0.0039*
27	Ig kappa chain C region	IGKC_HUMAN	P01834	12	1.80±0.74	1.05±0.35	1.72±0.51	0.5469
28	Ig lambda chain C regions	LAC_HUMAN	P01842	11	0.98±0.32	0.30±0.20	0.25±0.16	0.0212*
29	Ig mu chain C region	IGHM_HUMAN	P01871	49	0.70±0.26	0.93±0.33	1.14±0.24	0.3452
30	Inter-alpha-trypsin inhibitor H2	ITIH2_HUMAN	P19823	106	0.14±0.14	0.69±0.37	1.88±0.79	0.1126
31	Inter-alpha-trypsin inhibitor H4	ITIH4_HUMAN	Q14624	103	7.55±1.51	0.90±0.45	2.36±1.45	0.0008*
32	Lipopolysaccharide-binding protein	LBP_HUMAN	P18428	53	0.53±0.27	2.46±0.54	2.64±0.36	0.0008*
33	Platelet basic protein	CXCL7_HUMAN	P02775	14	0.65±0.34	0	0	0.0074*
34	Platelet factor 4	PLF4_HUMAN	P02776	11	1.20±0.64	0	0.25±0.16	0.0217*
35	Prothrombin	THRB_HUMAN	P00734	70	1.04±0.56	0	0	0.0093*
36	Serum albumin	ALBU_HUMAN	P02768	69	7.45±0.96	5.40±1.05	8.44±1.22	0.7226
37	Serum amyloid A protein	SAA_HUMAN	P02735	14	0.98±0.47	3.83±0.30	5.59±1.21	0.0016*
38	Serum amyloid A-4 protein	SAA4_HUMAN	P35542	15	0.29±0.18	0.25±0.16	0.20±0.20	0.7820
39	Sex hormone-binding globulin	SHBG_HUMAN	P04278	44	0	0	0.45±0.23	0.2520
40	Thrombospondin-1	TSP1_HUMAN	P07996	129	0.52±0.37	0	0	0.0377*
41	Transthyretin	TTHY_HUMAN	P02766	16	1.44±1.02	0.74±0.36	1.81±0.57	0.8472
42	Vitronectin	VTNC_HUMAN	P04004	54	7.35±0.89	5.59±1.26	6.07±0.91	0.2500

The normalized spectrum counts shown is mean of seven control, eight mild malaria and eight severe malaria samples for each protein identified, as calculated by Scaffold. P values indicate comparison of control versus malaria (mild and severe).

*Indicates significant difference between control and malaria patients (*P*<0.05). MW: molecular weight.

**Figure 3:**
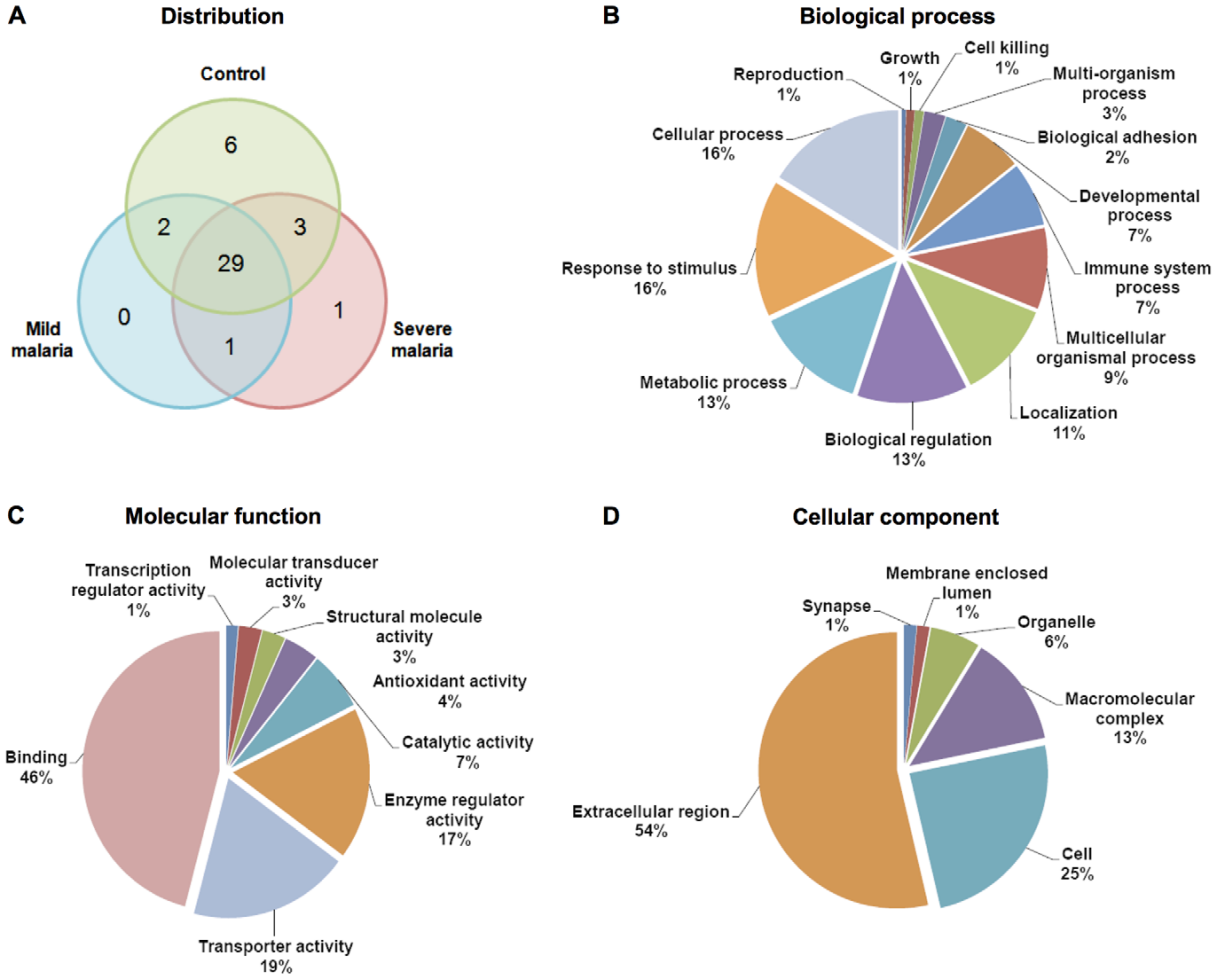
The distribution of hemozoin-binding proteins among the different groups and Gene Ontology terms (level 2). The number of hemozoin-binding proteins identified in each group (healthy controls, mild malaria, and severe malaria) and the distribution of the proteins among the different groups is shown by Venn diagram (**A**). Sequences of hemozoin-binding proteins were blasted, mapped and annotated using Blast2GO (B2G). A total of 701 Gene Ontology (GO) terms for biological process were identified, of which the distribution of the major ones is shown as biological process (**B**), molecular function (**C**) and cellular component (**D**).

The total number of unique peptides identified for each protein in the mild, severe, and healthy groups was used to compare the presence and abundance of each protein among the different groups. The distribution of the 42 HZ-binding proteins among the different groups (mild, severe and control) was also calculated. The distribution of the HZ-binding proteins identified in each group and the overlap between the different groups (control, mild and severe malaria) is shown in Figure 3A using Venn diagram. From this distribution analysis we did not observe a major difference between mild or severe malaria, at the exception of hemoglobin subunits alpha and beta. Most of the proteins (29 proteins) overlap between control and malaria group, two between control and mild, and three between control and severe malaria. Based on this distribution subsequent analysis was based on control and malaria groups.

### Functional analysis of HZ-binding proteins

In addition to distribution and abundance, functional analysis of the hemozoin-binding proteins was performed using Blast2GO (B2G), which is a comprehensive bioinformatics tool for functional annotation and analysis of protein sequences. The B2G analysis allowed us to elucidate the different functions and process in which the HZ-binding proteins are putatively involved. The 42 identified proteins were classified by their cellular (Figure 3B) and molecular function (Figure 3C) as well as by their cellular component (Figure 3D). Concerning cellular function, the identified proteins were involved in cellular process (16%), response to stimulus (16%), metabolic process (13%), biological regulation (13%) and localization (11%). Interestingly, according to the molecular function analysis (Fig. 3C), most of the proteins bound to HZ were related with binding (46%), transport (19%) or enzyme regulator activity (17%). In addition, most of the HZ-binding proteins were found in the extracellular (54%) or cell region (25%) as shown in Figure 3D by cellular component analysis.

**Figure 4:**
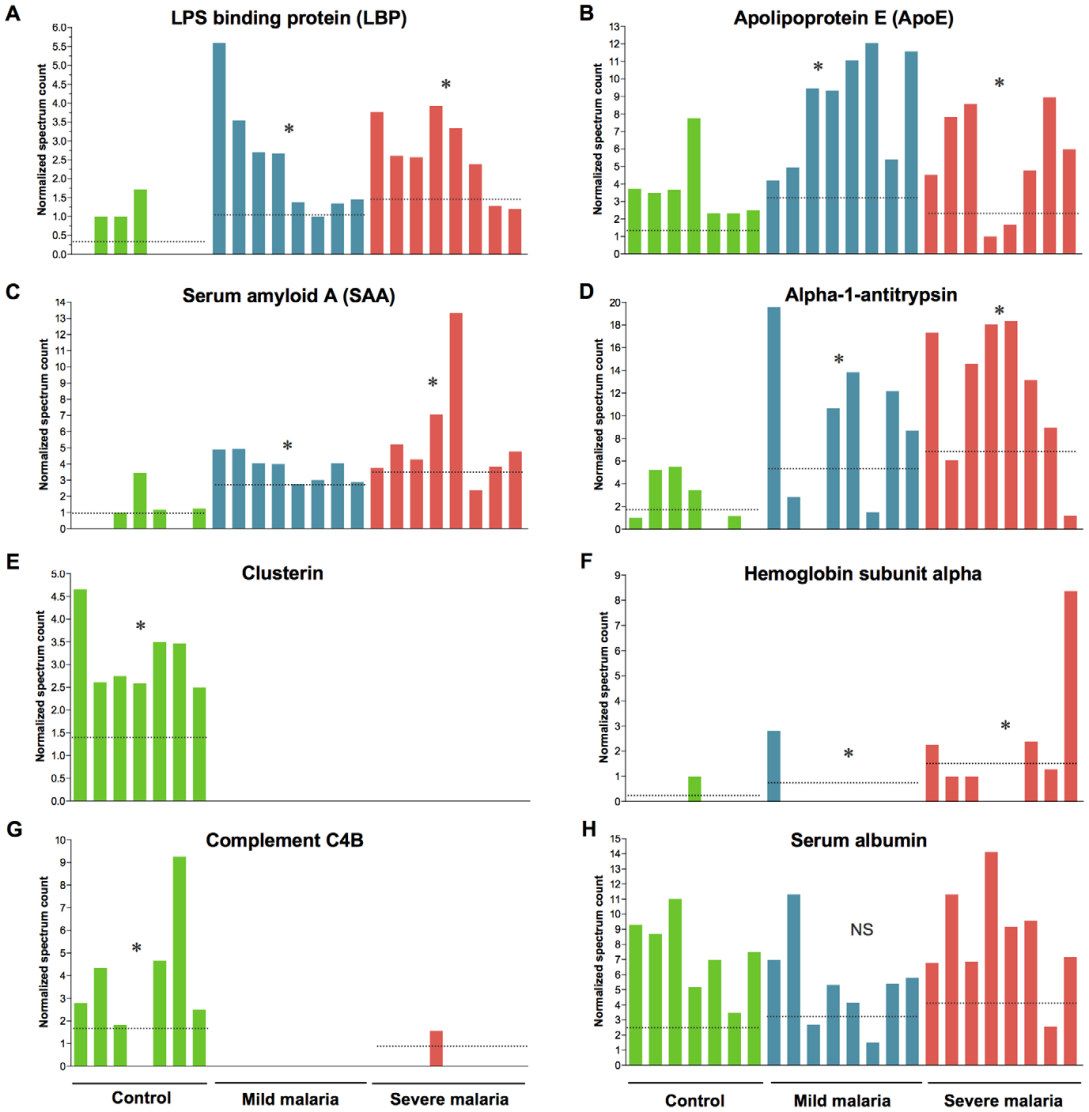
Normalized spectrum counts for selected hemozoin-binding proteins. Normalized spectrum counts, depicting the protein's relative abundance, is shown for selected hemozoin-binding proteins. **A**) LPS binding protein (LBP); **B**) Apoliporotein E (ApoE); **C**) serum amyloid A (SAA); **D**) alpha-1-antitrypsin; **E**) Clusterin; **F**) hemoglobin subunit alpha; **G**) Complement factor C4B and **H**) serum albumin shown as a control. LBP, ApoE, SAA, hemoglobin subunit alpha and alpha-1-antitrypsin were found in higher level in malaria patients whereas clusterin and complement C4B were present only in control samples. *P<0.05 for control vs. malaria (mild and severe).

Apart from the distribution of the identified HZ-binding proteins, further analysis was performed to compare their relative abundance across the different samples. For this we used the normalized spectrum counts of each identified protein and showed in histogram graphs for selected proteins bound to HZ. As shown in Figure 4, the peptides of LBP (Figure 4A), apolipoprotein E (Figure 4B), serum amyloid A (Figure 4C), alpha-1-antitrypsin (Figure 4D) and hemoglobin subunit alpha (Figure 4F) were found to be significantly higher in samples from malaria patients. On the other hand, clusterin (Figure 4E) and complement C4B (Figure 4G) were found only in the serum from control group. As expected, serum albumin is evenly distributed (Figure 4H). This analysis confirms the distribution of the proteins that could overlap between control and malaria group, however the abundance the proteins can be different according to the sample and group.

### Validating the association of malaria biomarkers with hemozoin

**Figure 5:**
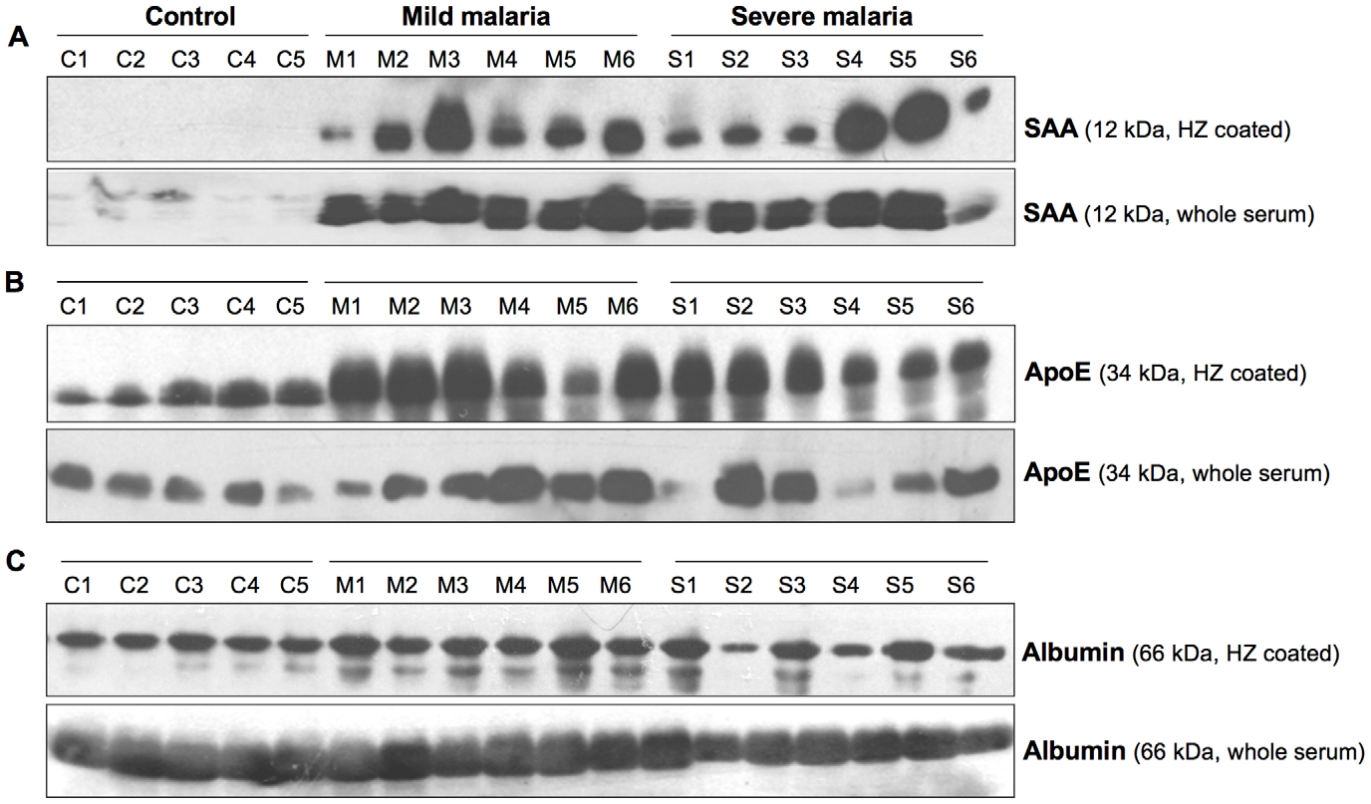
Western blots for selected biomarkers. Sera from mild and severe malaria patients and healthy individuals were either coated with hemozoin or run onto gel directly for western blot analysis. The membranes were blotted using specific antibodies for **A**) Serum amyloid A (SAA), **B**) Apolipoprotein E (ApoE) and **C**) serum albumin C1–C5: control; M1–M6: mild malaria; S1–S6: severe malaria.

To confirm the mass spectrometric findings, selected HZ-binding biomarkers and whole serum were subjected to Western Blotting analysis. The membranes were blotted using specific antibodies for serum amyloid A (SAA), apolipoprotein E (ApoE) and serum albumin. As can be seen from Figure 5, the contact of HZ with sera from malaria patient allows a higher binding with SAA (Figure 5A) and ApoE (Figure 5B) compared to the controls. The enhanced binding of these proteins to HZ might be related to their concentration in the serum as whole serum showed the same profile of SAA and ApoE. As control of protein binding and loading, we used serum albumin. As shown in Figure 5C, albumin was equally detected in the binding or whole sample from control or malaria patients.

**Figure 6:**
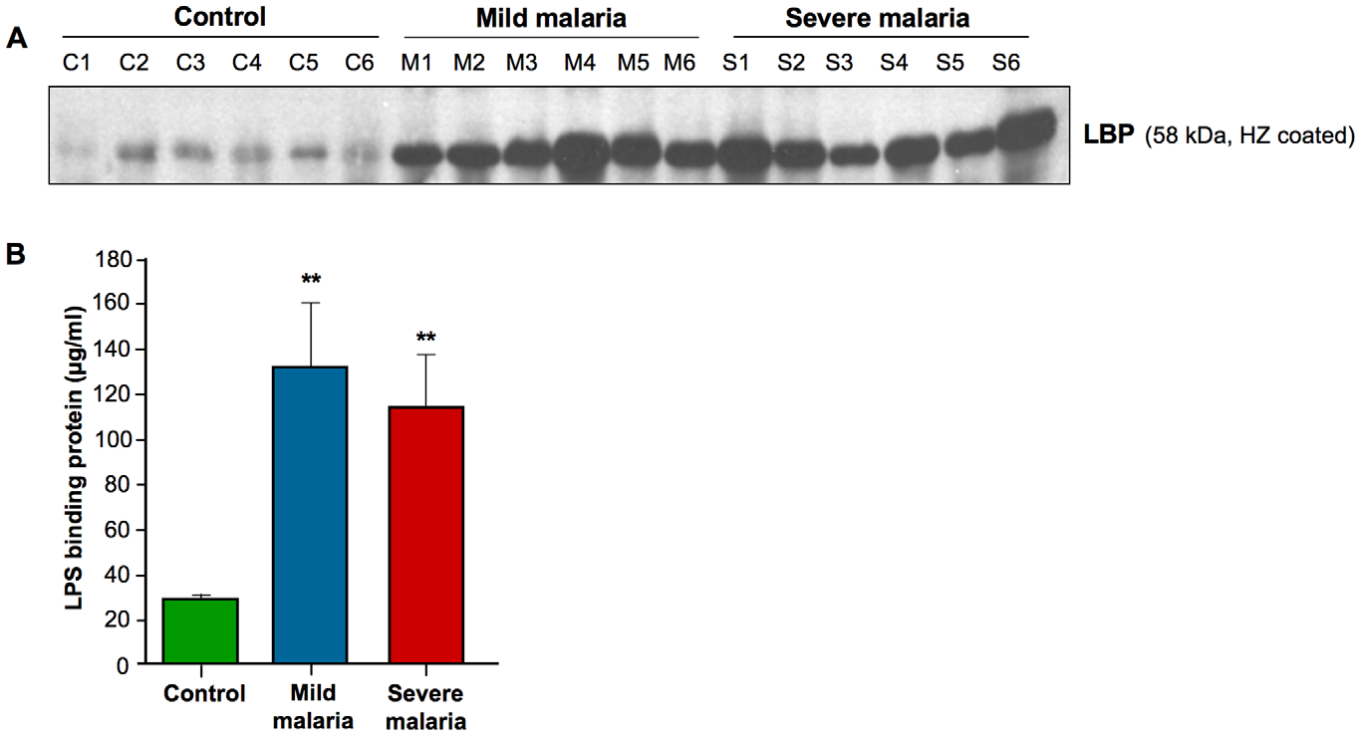
Confirmation of LPS binding protein using ELISA. **A**) Sera from mild and severe malaria patients and healthy individuals were coated with hemozoin and run onto gel for western blot analysis. **B**) The level of LPS binding protein (LBP) from sera of six mild, six severe malaria patients and six healthy individuals was quantified using sandwich ELISA. **P<0.05 for control vs. mild and control vs. severe.

In addition to SAA and ApoE, we also performed the Western Blotting analysis to LBP. As shown in Figure 6A, HZ coated with sera from mild and severe malaria patients showed a higher level of LBP compared to control sera. The total level of LBP -directly in the whole sera without HZ binding- was also quantified using sandwich ELISA specific for human LBP. As shown in Figure 6B, the level of LBP measured was significantly increased in malaria patients. Interestingly, and in contrast to LBP, ApoE and SAA which are elevated in serum of malaria patient, gelsolin was only found in serum from healthy individuals and disappeared in the malaria samples (Figure S1), suggesting that disappearance of normal serum proteins during malaria could be advantageous to include in biomarker profile related to malaria infection, since this reflects the pathological condition.

This last set of experiments confirmed the findings from the mass spectrometric analysis. Furthermore, Western Blotting results further confirm that different proteins can bind to HZ and that their binding levels correlate with the amount of protein present in the serum, as revealed by the analysis of total protein. In addition, the immunochemical analysis further revealed that the identified biomarkers cannot differ substantially from severe and mildly infected patients -at the exception for hemoglobin subunits alpha/beta that were clearly elevated in serum of severe malaria patients- but that it can clearly permit the discrimination between malaria and healthy individuals as depicted by the peptide distribution (Figure 4) and statistic analysis (Table 1).

## Discussion

In the present work, we identified new inflammation related biomarkers from the sera of malaria patients. Hemozoin, due to its amphiphilic characteristic, has been described to bind to CpG DNA [19] and proteins from the fetal bovine serum [13], [14]. We devised a novel way of screening potential biomarkers from the serum of malaria patients and healthy individuals using the malaria HZ crystal to trap these biomarkers. In this way, we have not only identified new malaria-related biomarkers but also potential co-factors that concomitantly with HZ could play a role in the modulation of inflammatory and innate immune responses during malaria.

Previous works explored biomarkers for malaria infection, particularly serum amyloid A (SAA) and C reactive protein (CRP) were identified as important malaria biomarkers [20]. In fact, SAA along with CRP has been proven to be valuable in assessing the severity of malaria and particularly as a prognostic tool in following response to treatments [20]. Interestingly we also detected SAA binding with hemozoin, but not CRP. The absence of lipid-binding domain in CRP could explain its failure to interact with hemozoin. SAA is an acute phase protein whose level increases during infection. Indeed, our results show that, SAA levels increase in the serum of malaria patients. SAA is a multifunctional protein which is involved in cholesterol transport and metabolism as well as inflammatory response [21].

The level of Apolipoprotein E (Apo E) also markedly increased in malaria patients. ApoE binds to lipids to form molecules called lipoproteins and known to be in part responsible for packaging cholesterol and other fats carrying them through the bloodstream. ApoE is a major component of a specific type of lipoprotein called very low-density lipoproteins (VLDLs) which remove excess cholesterol from the blood and carry it to the liver for processing. ApoE also serves as a ligand for low density lipoprotein receptors (LDLR) and ApoE receptor expressed in the hepatocytes (liver cells) [22]. Whether ApoE plays a critical role to tame malaria-related inflammatory responses or to detoxify the system from HZ, is still to be investigated.

More importantly, we report herein for the first time the up-regulation of LPS binding protein (LBP) in malaria. This latter is usually produced and secreted by the liver in response to Gram negative bacteria infection and particularly to the bacterial endotoxin LPS. LBP binds and transfers LPS to Toll-like receptor-4 (TLR-4) triggering an important signaling cascades and activation of inflammatory and innate immune response. Importantly, the level of LBP has been used as an important biomarker in sepsis and septic shock patients, where its levels increase up to 10-fold. Interestingly, depending on its level, LBP can be detrimental to the host at low-levels whereas beneficial in high amounts by detoxifying the system from LPS. Our findings, in the context of malaria, also suggest that LBP in conjunction with other identified proteins can be used as an important malaria biomarker. Furthermore, this finding supports the idea that malaria inflammatory disorder is often comparable to septic shock syndrome.

Other biomarkers identified include alpha-1-antitrypsin, clusterin, hemoglobin subunit alpha/beta and the complement C4B. The level of alpha-1-antitrypsin, which is an inhibitor of serine proteases, increases in malaria patients. On the other side, clusterin -also known as Apolipoprotein J- is completely absent from the sera of malaria patients. The function of clusterin in the context of malaria diseases is not yet clear. However, it is known to be expressed in a variety of tissues and is able to bind to cells, membranes and hydrophobic proteins. Its interaction with hydrophobic proteins might explain its binding ability to hemozoin, whereas its disappearance from malaria patients can confer an important role in the profiling of biomarkers related to malaria.

As malaria is characterized by a large number of red blood cell destruction, the levels of hemoglobin subunit alpha and beta were significantly elevated in malaria patients. Furthermore, this difference is pronounced when comparing mild and severe malaria patients, which correlates perfectly well with the level of infected red blood cells and anemia. The destruction of erythrocytes results also in the release of actin into the plasma. The actin-scavenging protein called gelsolin clears the excess actin. However, if the rate of clearance of actin-gelsolin complexes exceeds its synthesis, lowering of gelsolin levels will follow. Apart from actin binding function, our findings showed that gelsolin also binds hemozoin. This suggests that hemozoin-gelsolin complexes may also contribute to the depressed gelsolin levels observed during malaria.

Finally, complement activation in malaria is a well-documented phenomenon. Previous studies have shown that complement components C1q, C4, C3 and C5a change in malaria patients. In line with previous findings [24], our results also confirm complement C1q consumption in malaria patients. Furthermore, depletion of complement C3 was reported in severe and uncomplicated malaria patients as compared to healthy controls [23]–[27]. Collectively, those last observations further reinforce the validity of our study with the goal to identify new malaria related biomarkers.

As far as the scientific literature is concerned, there has not been any report that identifies serum proteins interacting with HZ. Hence apart from the biomarker discovery, our approach also aids in revealing the identity of serum proteins interacting with HZ. Even though much is known about the effect of HZ in the host immune system (as outlined in detail in the Introduction section), the role of HZ-binding proteins in the recognition, immune modulation and physiological clearance of HZ is unrevealed. Hence, the identification of HZ-binding proteins will help in elucidating the complete role of this inorganic crystal in the human host during malaria pathology.

In conclusion, we have identified specific HZ-binding proteins from serum of malaria patients and healthy individuals that can be useful to develop new assay based on profile of malaria biomarkers. The role of HZ-binding proteins in the crystals' recognition by the immune system and its clearance remains to be investigated in a near future. Collectively, we believed that our actual study bring in a new devise that can be very helpful to concomitantly identify biomarkers related to malaria and to develop a specific biomarker-based diagnostic tool.

## Supporting Information

Figure S1 **Western blots for selected biomarkers.** Sera from malaria patients and healthy individuals were coated with hemozoin and run onto gel for western blot analysis. The membranes were blotted using specific antibodies for gelsolin, LPS binding protein (LBP), Serum amyloid A (SAA), Apolipoprotein E (ApoE) and serum albumin. C1–C8: control; M1–M8: malaria.
(TIF)
