# Supplementary figures and images for: New Inflammation-Related Biomarkers during Malaria Infection
Source: PLoS One. 2011 Oct 20;6(10):e26495. doi: 10.1371/journal.pone.0026495 (PMC3197653; doi:10.1371/journal.pone.0026495)

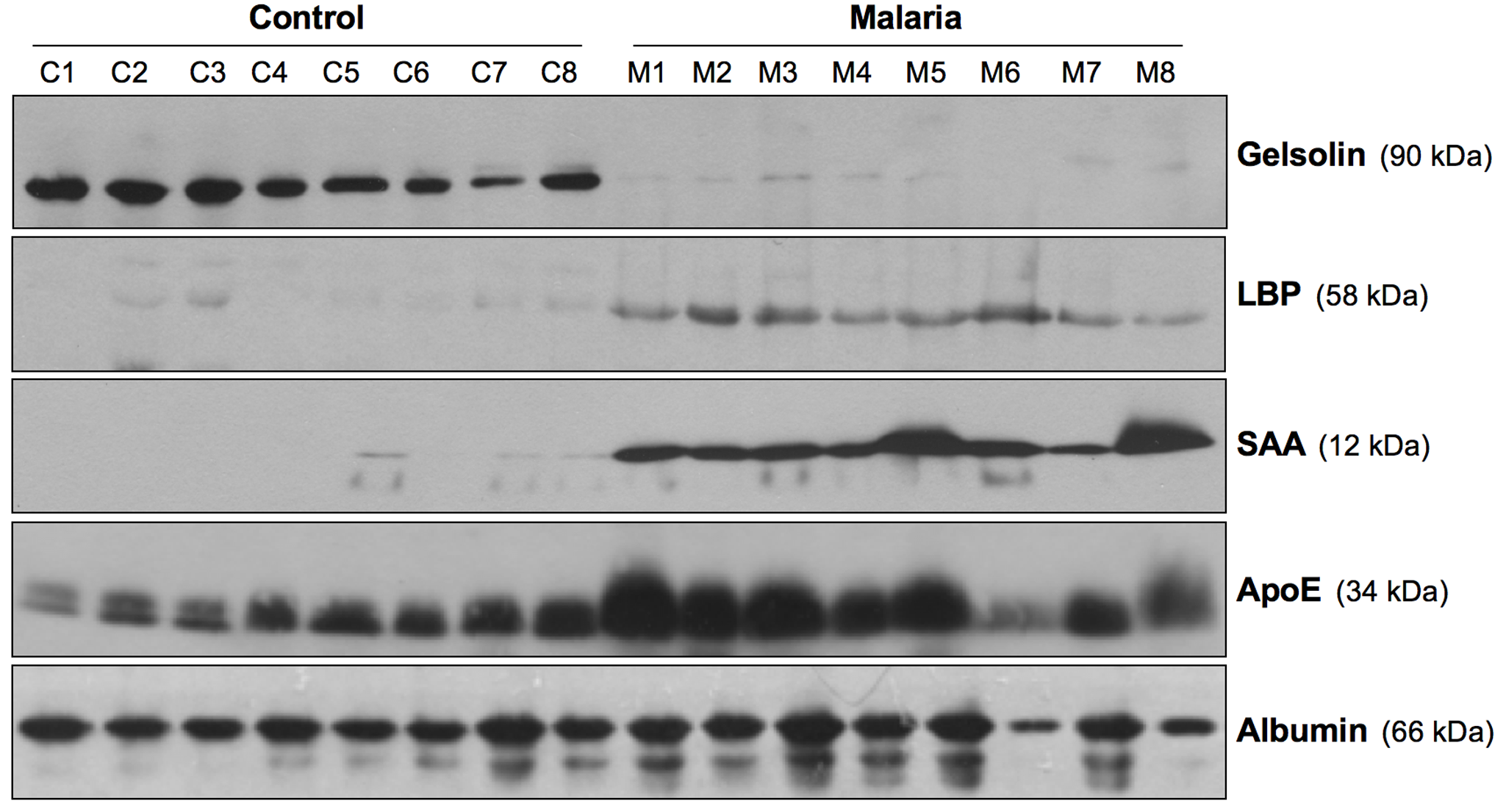

Supplement: Figure S1 — Western blots for selected biomarkers. Sera from malaria patients and healthy individuals were coated with hemozoin and run onto gel for western blot analysis. The membranes were blotted using specific antibodies for gelsolin, LPS binding protein (LBP), Serum amyloid A (SAA), Apolipoprotein E (ApoE) and serum albumin. C1–C8: control; M1–M8: malaria. (TIF) [file pone.0026495.s001.tif]
